# Factors contributing to delay intensive care unit admission of critically ill patients from the adult emergency Department in Tikur Anbessa Specialized Hospital

**DOI:** 10.1186/s12873-021-00518-z

**Published:** 2021-10-26

**Authors:** Helen Teklie, Hywet Engida, Birhanu Melaku, Abdata Workina

**Affiliations:** 1 AaBET hospital, Addis Ababa, Ethiopia; 2 Department of emergency medicine, Addis Ababa University, Addis Ababa, Ethiopia; 3 School of Nursing, Jimma University, Jimma, Oromia Ethiopia

**Keywords:** Critical patient, Emergency department, ICU admission, Delay

## Abstract

**Background:**

The transfer time for critically ill patients from the emergency department (ED) to the Intensive care unit (ICU) must be minimal; however, some factors prolong the transfer time, which may delay intensive care treatment and adversely affect the patient’s outcome.

**Purpose:**

To identify factors affecting intensive care unit admission of critically ill patients from the emergency department.

**Patients and methods:**

A cross-sectional study design was conducted from January 13 to April 12, 2020, at the emergency department of Tikur Anbesa Specialized Hospital. All critically ill patients who need intensive care unit admission during the study period were included in the study. A pretested structured questionnaire was adapted from similar studies. The data were collected by chart review and observation. Then checked data were entered into Epi-data version 4.1 and cleaned data was exported to SPSS Version 25 for analysis. Descriptive statistics, bivariate and multivariate logistic regression were used to analyze the data.

**Result:**

From the total of 102 critically ill patients who need ICU admission 84.3% of them had prolonged lengths of ED stay. The median length of ED stay was 13.5 h with an IQR of 7–25.5 h. The most common reasons for delayed ICU admission were shortage of ICU beds 56 (65.1%) and delays in radiological examination results 13(15.1%). On multivariate logistic regression *p* < 0.05 male gender (AOR = 0.175, 95% CI: (0.044, 0.693)) and shortage of ICU bed (AOR = 0.022, 95% CI: (0.002, 0.201)) were found to have a significant association with delayed intensive care unit admission.

**Conclusion:**

there was a delay in ICU admission of critically ill patients from the ED. Shortage of ICU bed and delay in radiological investigation results were the reasons for the prolonged ED stay.

**Supplementary Information:**

The online version contains supplementary material available at 10.1186/s12873-021-00518-z.

## Introduction

An emergency department (ED) is a hospital unit responsible for managing and stabilizing sick patients in need of immediate care, followed by a transfer to the appropriate health care provider, but when patients are critically ill and require intensive care and monitoring they must be admitted to the intensive care unit timely [1–5].

The transfer time of critically ill patients from the ED to the ICU must be short, according to the recommendations of the Society of Critical Care Medicine (SOCCM) [2]. The emergency medicine practitioners should be ready to provide critical care in the ED, considering the frequent unavailability of ICU beds. However, studies indicate that patients who meet the ICU admission criteria and are treated in the ICU had a higher survival rate than those treated out of the ICU [2, 6–8]. Duration of hospitalization before ICU admission is an independent predictor of ICU outcome [9, 10]. So, it is important to recognize critical illness early to enable the prompt transfer of patients who may benefit from the intensive care [11–13]. However, studies show that critical care in low-income countries remains a neglected field of health service provision, with large numbers of patients with potentially treatable conditions unable to receive these facilities [14–17]. Additionally, in Ethiopia, limited studies also show that critically ill patients spent a prolonged time in the ED and they had poor outcomes due to limited critical care services [14, 15].

Delays in transferring patients from the ED to the ICU can have a negative impact on patient outcomes, including ED overcrowding, increased hospital mortality and ICU length of stay as well as increased mechanical ventilation requirement during ICU stay [16, 18–22]. A study conducted in Pakistan shows**,** critically ill patients staying in the ED for more than 6 h have a 27.3% higher hospital mortality rate than those who were transferred early to the ICU. Furthermore, a study conducted in Ethiopia indicated that, amongst critically ill patients who have more than 6 h length of emergency department stay (EDLOS), 32.3% of them died before transferring to the ICU [15]. Some studies revealed that lack of critical care beds, delay in radiology and laboratory test services, and limited ICU resources were the common reasons which predisposed patients to prolonged emergency department stay [15–17, 21, 23, 24].

Therefore, this study was aimed to identify factors associated with delayed ICU admission.

## Patients and methods

### Study design and setting

An institutional-based cross-sectional study design was employed at Tikur Anbessa specialized hospital (TASH) from January 13 to April 12, 2020. TASH is the largest tertiary public hospital in Ethiopia and serves approximately 700,000 patients a year. The hospital is serving as a teaching and training center for health programs. The adult emergency department (ED) of TASH had provided services to approximately 18,000 patients per year. The adult ICU of TASH had 16 surgical ICU beds and 16 medical ICU beds with a total of 32 adult intensive care unit beds [25].

#### Study population

All critically ill patients who were admitted to the adult emergency department and needed intensive care unit admission during the data collection period at TASH.

### Eligibility criteria

All critically ill patients who consulted for ICU admission in the adult ED were included in the study while a patient chart that has inadequate information was excluded.

### Sample size determination and sampling procedure

All critically ill adult patients who were admitted to the ED and consulted for intensive care unit admission during the data collection period were included and the data collectors observed the admission process until the patient left the ED. The number of the less frequent outcome in logistic regression was 16 patients who had on time ICU admission which is small and might compromise the result. So, to address this we adopted ten events per variable (EPV) minimal guideline criterion for performing multivariate logistic regression analysis.

### Data collection tool and procedure

A structured questionnaire was adapted from up-to-date literature [15, 17, 21, 26, 27]. The questionnaires contain patient demographics, time of arrival to the ED, time of decision for ICU admission, working diagnosis, procedures performed, and time of patient disposition. Data was collected using medical record charts review and observation of critically ill patients who wait for intensive care unit admission until the patient left the ED. Data was collected by trained three emergency and critical care nurses working in the study hospital.

### Operational definition

Delayed intensive care unit admission is when a critically ill patient spent more than 6 h in the ED after consulting and deciding for ICU admission [15, 17, 28].

NEWS (National Early Warning Score): is an evidence-based system of care, used to facilitate timely recognition of patients with established or impending critical illness and allow for timely admission to intensive care, (NEWS ≥7 high score, NEWS > 5 medium scores and NEWS 1–4 low score) [28, 29].

### Data quality assurance

Questionnaires were pretested at AaBET hospital for 2 weeks and computed Cronbach’s alpha was 0.82. Data collectors were trained on the contents of questionnaires before data collection. The questionnaires were filled upon ED admission of critically ill patients who need ICU admission and completed during disposition with documentation of time of disposition. Each questionnaire was checked for completeness, and consistencies and incomplete questionnaires were omitted from the analysis.

### Data processing and analysis

The checked and coded data were entered into Epi-data version 4.1. Then, cleaned data was exported to SPSS version 25 for analysis. Descriptive statistics were computed to summarize categorical variables. ICU admission was dichotomized into delayed and not delayed. Model fitness was checked using Hosmer and Lemeshow goodness of fit model. A multicollinearity test was computed (SE < 2.0). Bivariate logistic regression was done to identify candidate variables for multivariate logistic regression at *P* < 0.25. Then, in multivariate logistic regression; predictors having *p*-value < 0.05 at CI 95% were considered statistically significant association.

### Ethical consideration

An ethical approval letter to conduct the study was obtained from the Addis Ababa University department of Emergency Medicine ethical review committee with the Ref No: EM/SM/344/2012. A letter was given to the TASH administrator for permission to conduct the study. The research purpose, its benefits, and the procedures were explained for the Emergency Department staff member and manager. Confidentiality and privacy were strictly maintained. Since the data was collected by patients chart review Addis Ababa university department of emergency medicine ethical review committee waived participants’ consent. Only the principal investigator and the research assistants can access the data. All methods throughout the study were performed in accordance with the relevant guidelines and regulations.

## Results

### Socio-demographic characteristics

A total of 102 critically ill patients who needed and consulted for ICU care were observed during the data collection period and their recorded data were collected. The mean age of patients was 40.4 ± 17.7 SD years. Among the study participants, 61 (59.8%) of them were males. Regarding their source of referral, the majority of critically ill patients 53 (52%) had come from another governmental hospital. (Table 1).

**Table 1:** Socio-demographic characteristics and baseline information of critically ill patients at TASH adult ED, Addis Ababa, Ethiopia, 2020

Variables			Frequency (*n* = 102)	Percent (%)	ICU admission	
Age group (years)	Mean ± SD	40.4 ± 17.7			Prolonged	On-time
	< 18		8	7.8	6 (7.0%)	2 (12.5%)
	18–50		59	57.8	50 (58.1%)	9 (56.3%)
	> 50		35	34.3	30 (34.9%)	5 (31.3%)
Sex	Male		61	59.8	55 (64.0%)	6 (37.5%)
	Female		41	40.2	31 (36.0%)	10 (62.5%)
Residence	Addis Ababa		62	60.8	50 (58.1%)	12 (75.0%)
	Oromia		26	25.5	24 (27.9%)	2 (12.5%)
	Other^a^		14	13.7	12 (14.0%)	2 (12.5%)
Source of referral	Self		34	33.3%	26 (30.2%)	8 (50.0%)
	Another governmental hospital		53	52.0%	46 (53.5%)	7 (43.8%)
	Others^b^		15	14.8	14 (16.3%)	1 (6.3%)
Trauma	Yes		10	9.8	10 (11.6%)	0 (0.0%)
	No		92	90.2	76 (88.4%)	16 (100.0%)
NEWS score	5–6		10	9.8	9 (10.5%)	1 (6.3%)
	≥ 7		92	90.2	77 (89.5%)	15 (93.7%)
Past medical illness(*n* = 102)	Yes		75	73.5	62 (72.1%)	13 (81.3%)
	No		27	26.5	24 (28.0%)	3 (18.8%)
Types of past medical illness (*n* = 75)	Chronic kidney disease		4	5.3	4 (4.7%)	0 (0.0%)
	Chronic glomerulonephritis		3	4.0	1 (1.2%)	2 (12.5%)
	Renal stone		2	2.7	2 (2.3%)	0 (0.0%)
	Chronic valvular heart disease		8	10.7	7 (8.1%)	1 (6.3%)
	Chronic atrial fibrillation		4	5.3	2 (2.3%)	2 (12.5%)
	Congestive heart failure		5	6.7	3 (3.5%)	2 (12.5%)
	Coronary artery disease		9	12.0	6 (7.0%)	3 (18.8%)
	Dilated cardiomyopathy		2	2.7	2 (2.3%)	0 (0.0%)
	Rheumatic heart disease		4	5.3	4 (4.6%)	0 (0.0%)
	Esophageal carcinoma		1	1.3	0 (0.0%)	1 (6.3%)
	Chronic myeloid leukemia		4	5.3	2 (2.3%)	2 (12.5%)
	Non-Hodgkin lymphoma		8	10.7	6 (7.0%)	2 (12.5%)
	Breast cancer		3	4.0	3 (3.5%)	0 (0.0%)
	Colorectal cancer		5	6.7	4 (4.7%)	1 (6.3%)
	Tracheobronchial tumor		1	1.3	0 (0.0%)	1 (6.3%)
	Thyroid cancer		1	1.3	1 (1.2%)	0 (0.0%)
	Bladder cancer		1	1.3	1 (1.2%)	0 (0.0%)
	Others^c^		10	13.3	7 (8.1%)	3 (18.8%)
Managements provided at TASH emergency department **Notes:** multiple answers were possible	Non-invasive ventilator support		67	70.5	62 (72.1%)	5 (31.5%)
	Endotracheal intubation		21	22.1	19 (22.1%)	2 (12.5%)
	Both non-invasive ventilator support and endotracheal intubation		7	7.4	4 (4.6%)	3 (18.8%)
	Initiation of broad-spectrum antibiotic		87	85.3	75 (87.2%)	12 (75.0%)
	Initiation of vasopressor		34	33.3	27 (31.4%)	7 (43.8%)
	On monitoring		102	100.0	86 (100.0%)	16 (100.0%)
	Cardiopulmonary Resuscitation		6	5.9	5 (5.8%)	1 (6.3%)
Diagnosis at the red zone of ED	Trauma		7	6.9	7 (8.1%)	0 (0.0%)
	Septic shock		30	29.4	24 (27.9%)	6 (37.5%)
	Acute respiratory failure requires ventilator support		34	33.3	30 (34.9%)	4 (25.0%)
	Acute kidney injury		7	6.9	6 (7.0%)	1 (6.3%)
	Ventricular tachycardia		6	5.9	4 (4.6%)	2 (12.5%)
	Ventricular fibrillation		2	1.9	2 (2.3%)	0 (0.0%)
	Others**$**		16	15.7	13 (15.1%	3 (18.8%)

Other^a^; Amhara, SNNPR, Tigray & Afar, Others^b^; Private health facility, Public health centre, others^c^; Endocrine, Respiratory, Gastrointestinal, Central nervous system, others$; Stroke, meningitis, other types of shock, Neutropenic fever and Tetanus

### Baseline information of critically ill patients

From the total critically, ill patients who consulted for ICU admission 92 (90.2%) were non-trauma patients while the rest were trauma patients. The majority of 92 (90.2%) of the critically ill patients who consulted for ICU care, were high-risk patients with NEWS was ≥7. Most patients 75 (73.5%) were also having a known chronic medical illness, whereas 27(26.5%) had no known chronic medical illness. Among patients with past medical illness, the majority 32(42.7%) had cardiovascular illness followed by malignancies 24 (32.0%). (Table 1).

### Management has given for critically ill patients and reason for red ward admission

The majority of critically ill patients 95 (93.1%) were on ventilator support, from those 21 (22.1%) were on a mechanical ventilator through endotracheal intubation and the rest, 67(70.5%) were on non-invasive ventilation, while 7(7.4%) of them had received both non-invasive ventilator support and endotracheal intubation. Most of the patients 87 (85.3%) were started with a broad-spectrum antibiotic and those who were admitted due to shock 34 (33.3%) were on vasopressor in the ED and all patients were on monitoring. The most critically ill patients’ diagnosis at the red zone of ED was acute respiratory failure requiring ventilator support 34(33.3%), followed by Septic shock 30(29.4%). (Table 1).

### Critically ill patients length of stay at ED and reasons for delay ICU admission

Among critically ill patients who consulted for ICU admission, 86 (84.3%) of them had stayed for more than 6 h in the ED before transfer to ICU or they had a delayed ICU admission. Of the 102 critically ill patients who needed and consulted for critical care 53 (52%) were transferred and 49 (48%) were not transferred to the ICU during the study period. The lengths of ED stay ranged from 1 h to 144 h. Most critically ill patients 35 (34.3%) had an emergency department length of stay of 6–12 h. The median length of stay was 13.5 h, with an IQR of 7–25.5 h. Of patients who had been delayed for ICU admission, 30(34.9%) of them died while waiting for ICU admission,37(43.0) of them were admitted to ICU after delayed for ICU admission and 11(12.8%) patients were admitted to inpatient ward whereas 8(9.3%) of them improved and discharged from ED. Among those patients who had delayed ICU admission the most reason for the delay was due to lack of ICU bed 56(65.1%), followed by a delay in radiological investigations result 13(15.1%), whereas a delay in the therapeutics procedure done in the ED was the least factor contributing to delay ICU admission. (Table 2).

**Table 2:** Lengths of ED stay of critically ill patients who need ICU admission and reasons for delay ICU admission at TASH, Addis Ababa, Ethiopia, 2020

Variables		Frequency	Percent
ED lengths of stay(*n* = 102)	< 6 h.	16	15.7
	6–12 h.	35	34.3
	13–24 h.	25	24.5
	25–48 h.	10	9.8
	> 48 h.	16	15.7
Median	13.5 h.		
IQR	7–25.5 h.		
Critically ill patients who need and consulted for critical care were transferred to the ICU	Yes	53	52
	No	49	48
Outcomes of prolonged ICU admission(*n* = 86)	Death	30	34.9
	Admitted to ICU after delayed for ICU admission	37	43.0
	Admitted to inpatient ward	11	12.8
	Improved and discharged from ED	8	9.3
**Column1** **Column2** Reasons for delay ICU admission(*n* = 86)	Lack of ICU bed	56	65.1
	Delay in radiological result	13	15.1
	Poor prognosis	8	9.3
	Delay of lab. Investigation results	6	7.0
	Delays in therapeutics	3	3.5

### Factors associated with delayed ICU admission of critically ill patients

Bivariate and multivariate logistic regression analyses were performed to identify the existence of an association between the delayed ICU admission and Socio-demographic characteristics, patient-related factors, and organizational factors. In bivariate logistic regression analysis, the factors found to be significantly associated with delayed ICU admission with a *p*-value < 0.25 were the lack of ICU bed, sex, having malignancy, and cardiovascular disease.

On the other hand, to control the effect of confounding variables, factors with *p* value< 0.25 were entered into multivariate logistic regression and lack of ICU bed and male gender was statistically significant to predict delayed ICU admission in the multivariate logistic regression with a *p*-value of < 0.05.

In a binary logistic, patients who had a history of cardiovascular disease were 3.08 times higher to transfer in the ICU with less than 6 h than those who don’t have the disease (COR 3.08 (0.75–6.80) but patients who had malignancy were less likely to transfer to the ICU with less than 6 h by 65% than who don’t have the disease (COR = .35(0.03, 1.77)).

However, in multivariate regression, only male gender and lack of ICU bed had an association. Male gender critically ill patients were less likely to transfer to the ICU within less than 6 h by 82% than females (AOR = 0.18, 95% CI: (0.04, 0.69)) and when there was a lack of ICU bed, transfer to the ICU in less than 6 h ED stay were less likely by 97% (AOR = 0.03, 95% CI: (0.02, 0.20)). (Table 3).

**Table 3:** Bivariate and Multivariate logistic regression analysis of factors associated with delayed ICU admission of critically ill patients at adult ED of TASH, Addis Ababa, Ethiopia, 2020

Variables Category		ICU admission		*P*-value < 0.25	COR (95%CI)	*P*-value < 0.05	AOR (95%CI)
		Not prolonged ED stay	Prolonged ED stay				
Age	≤50	11	56	.465	1.80 (.91,7.84)		
	> 50	5	30		1		
Sex	Male	6	55	.054*	.34(.11,1.02)	.013**	.175(.044,.693)
	Female	10	31		1		
Lack of ICU bed	Yes	1	55	.002*	.034(.005,.298)	.001**	.022(.002,.201)
	No	15	31		1		
Delay of investigations result	Yes	4	12	.999	1.46 (0.86, 5.23)		
	No	16	70		1		
Delay in therapeutic procedure	Yes	11	6	.999	9.30 (3.49, 16.31)		
	No	14	71		1		
Poor prognosis	Yes	9	13	.999	3.92 (1.23, 9.48)		
	No	12	68		1		
CVD	Yes	10	22	.146*	3.08(.75,6.79)	.522	1.58(.39,6.3)
	No	9	61		1		
Malignancy	Yes	2	22	.155*	.35(.03,1.77)	.132	.16(.02,1.73)
	No	16	62		1		
Renal	Yes	2	16	.575	0.65 (.30,8.57)		
	No	14	73		1		

Crude odds ratio (COR) = *P < 0.25; Adjusted odds ratio (AOR) = **P < 0.05, *CI* Confidence interval, *CVD* Cardiovascular disease, *ICU* Intensive care unit, *ED* Emergency department

## Discussion

Emergency Department Length of stay is considered a key measure of emergency department throughput, and it is perceived as a measure of healthcare service quality, especially for those who need ICU care [30]. The society of critical care medicine (SOCCM) 2016, suggests the transfer time of critically ill patients from the ED to the ICU should be minimized or < 6 h. So, the aim of this study was to identify factors that affect ICU admission of critically ill patients and their length of stay in the ED. The result of the current study revealed that 86 (84.3%) of the critically ill patients had been delayed for ICU admission, while only the rest patients, 16 (15.7%), were transferred to the ICU in less than 6 h of ED stay. This was comparable to the same study done in Pakistan in which 67.7% of them stayed in the ED for more than 6 h before transferred to the ICU [17]. The results were also relatively comparable with a study conducted in Ethiopia among 431 critically ill patients who need ICU admission; the results reported that around 67.5% of the patients had delayed ICU admission [15], This number discrepancy might occurred due to that, this previous study conducted in Ethiopia was included the pediatric ED patients, besides sociodemographic characteristics of patients might also change over time. This shows that critical care service in Ethiopia still needs an improvement.

In contrast to these studies, a study conducted in Finland shows EDLOS of critically ill patients was short and from the total critically ill patients, 79.3% admitted to the ICU within 3 h of ED admission [21], the possible reason for this study finding gap was due to difference between low income and high-income countries healthcare access. Additionally, this study shows that the median time of EDLOS was 13.5 h, and this demonstrates still there is a prolonged emergency department length of stay of critically ill patients as the emergency time target was < 6 h. This study was relatively consistent with the study conducted in Pakistan which reveals, the median emergency department LOS of critically ill patients who need ICU care was 10.5 h [17], and a similar study conducted in Ontario, Canada showed the median EDLOS for all ICU admissions from ED was 7 (4–13) hours [26]. In contrast to these studies, a study conducted in Ethiopia in 2016 showed that the median EDLOS was 48 h [15]. These result discrepancies may be the result of some improvement in the health sector; few governmental and nongovernmental health facilities were launched.

High EDLOS may lead to increased ED overcrowding and may have an impact on the critically ill patient outcome, whereas certain organizational resource allocation and critical care service improvement may have a positive effect on it. Interdisciplinary methods can be utilized to investigate factors causing prolonged EDLOS and contribute a better understanding of them.

Based on the data presented in this study, an acute respiratory failure that requires ventilator support 34(33.3%), was the common reason for the need for ICU admission and septic shock was the second 30(29.4%), followed by Life-threatening dysrhythmias 7.8%, but a study was done in Ontario Canada and Pakistan showed cardiovascular disease is the most common cause of ICU admission (36 and 47.6% respectively) [17, 26], this discrepancy may be because cardiovascular disease is the leading cause of mortality especially in the developed countries. In the previous study done in Ethiopia and also in Finland trauma was the common reason for ICU admission (11.6 and 21.1% respectively) [15, 21], which was not comparable to the current study. This may be due to trauma centers being launched in the city after the studies were conducted.

There are many major contributing factors for delayed ICU admission and in this study, nearly half 56 (54.9%) of critically ill patients had delayed for ICU admission due to lack of ICU bed 56 (65.1%) followed by delays in radiological investigation results 13(15.1%) and poor prognosis, delayed laboratory investigation results, and delayed in therapeutic procedures that can be done in the ED was the reasons (8(9.3%), 6 (7.0%), and 3 (3.5%)) respectively. This study goes in line with a previous study done in Ethiopia which revealed lack of ICU beds is the main reason for the prolonged ED stay [15]. While studies conducted in Finland and Pakistan showed diagnostic and therapeutic procedures that can be done in the ED and diagnostic group was the main reason for those who have delayed ICU admission [17, 21], it shows their ICU bed capacity was better than ours.

This study indicates that critical care service is limited in the study hospital and other facilities like radiological service and laboratory service need improvement. Patients who had malignancy and those who had severe illness spent prolonged ED stay due to their poor prognosis and scarcity of critical care units the physician prioritizes other patients.

The present study reveals critically ill patients who had co-morbidity have delayed ICU admission, but in multivariate analysis, it doesn’t show any significant association. This finding was consistent with study conducted in Madrid, Spain which reveals that patients with co-morbidly was the risk factor associated to prolongation of ED length of stay [31]. Male critically ill patients were less likely to transfer to the ICU within 6 h of ED stay by 82% than females (AOR =0.175(0.044, 0.693)). This was consistent with a retrospective study conducted in Finland that reveals gender of study participants had association with prolonged ICU admission (*P* = 0.004) [21]. This study is not comparable with the study done in Ontario, Canada, which shows no significant difference was found between male and female critically ill patients regarding delayed ICU admission [26]. This may be possibly due to co-morbidity diseases like cancer having a higher prevalence in males than females.

This study reveals that lack of ICU bed (AOR = 0.022, 95% CI: (0.002,0.201)) has a significant association with delayed ICU admission and it shows that critically ill patients were less likely to transfer with less than 6 h by 97% when there is lack of ICU bed. This study was consistent with a study conducted in Turkey which shows, lack of space in the intensive care unit had a significant association for delayed ICU admission [12]. Furthermore, this study was supported with another study conducted in Ethiopia, which shows shortage of ICU beds were significant association with delayed ICU admission(AOR = 8.7, 95%CI:(3.2–23.2)) [16].

This contradicts a study done in Finland, which shows a performed radiological investigation was scored significantly higher than the other factors that cause delayed ICU admissions [21]. These observations could be explained by the fact that even though the scarcity of ICU beds were a common worldwide problem, this issue was the extreme problem in developing countries, which contributes for delayed ICU admission for critically ill patients that had negative effect on patient outcomes as our study shows that, among patients who had been delayed for ICU admission, 30(34.9%) of them died while waiting for ICU admission.

To improve timely ICU admission of critically ill patients the organization should have to more IICU beds, train the ED doctors about prioritizing transfer over radiology exams, and transfer the investigation results of critically ill patients to ED timely.

### Strengths and limitations of the study

The limitations of the study were, as the study was based on a single institution, generalization as a whole might be not considered. Besides, a cross-sectional study by its nature cannot establish a definitive cause and effect relationship to identify the risk factors. Additionally, even though the finding of the study was interesting, due to small sample size generalization of outcome patients who had prolonged ICU admission was difficult.

The strengths of this study were that variables were categorized and used based on ICU admission guidelines. The study data added the outcomes of delayed ICU admission and the reason for prolonged ICU admission from ED of critically ill patients which can be a source especially for low income countries. .

## Conclusion

The majority of the critically ill patients spend prolonged time in the ED at TASH despite requiring ICU admission. Lack of ICU bed, delays in the radiological examination, and laboratory investigation services were the most important factors which lead to delayed transfer to ICU. Also, the majority of critically ill patients need intensive care unit transfer due to acute respiratory failure that requires ventilator support. In the multivariate logistic regression being male gender and lack of ICU bed were predictors of delayed ICU admission of critically ill patients.

## Supplementary Information

**Additional file 1.**

## Data Availability

The datasets generated and analyzed during the current study are not publicly available due to these data were used under license for the current study but are available from the corresponding author on reasonable request.
