# Supplementary material for: Factors contributing to delay intensive care unit admission of critically ill patients from the adult emergency Department in Tikur Anbessa Specialized Hospital
Source: BMC Emerg Med. 2021 Oct 26;21:123. doi: 10.1186/s12873-021-00518-z (PMC8547562; doi:10.1186/s12873-021-00518-z)
Supplement: Supplementary file 1 — Additional file 1. [file 12873_2021_518_MOESM1_ESM.docx]

**Appendix**

| **National Early Warning Score (NEWS)** | | | | | | | |
| --- | --- | --- | --- | --- | --- | --- | --- |
| Physiological parameters | 3 | 2 | 1 | 0 | 1 | 2 | 3 |
| Respiration rate | ≤8 |  | 9-11 | 12-20 |  | 21-24 | ≥25 |
| Oxygen saturation | ≤91 | 92-93 | 94-95 | ≥96 |  |  |  |
| Any supplemental oxygen |  |  | Yes | No |  |  |  |
| Temprature(in °C) | ≤35.0 |  | 35.1-36.0 | 36.1-38.0 | 38.1-39.0 | ≥39.1 |  |
| Systolic BP | ≤90 | 91-100 | 101-110 | 111-219 |  |  | ≥220 |
| Heart rate | ≤40 |  | 41-50 | 51-90 | 91-110 | 111-130 | ≥131 |
| Level of consciousness |  |  |  | A |  |  | V,P or U |

<https://www.rcplondon.ac.uk/projects/outputs/national-early-warning-score-news-2>

**Interpretation**

- A low score (NEWS 1–4) - Prompt assessment and frequent of clinical monitoring or an escalation of clinical care is required.
- A medium score (NEWS of 5–6 or a RED score) - Escalation of care to a team with critical-care skills is required.
- A high score (NEWS ≥7) - Prompt transfer of the patient to a higher dependency care area.
